# Intestinal obstruction due to rectal neoplasia in a patient with ulcerative colitis undergoing surgery with total mesorectal excision: case report and literature review

**DOI:** 10.1097/RC9.0000000000001088

**Published:** 2026-09-29

**Authors:** Maria Stella Vasconcelos Sales Valente, Mayara Marina Sousa Senzano, Lívia Moreira Genaro, Luisa Souza Echeverria, Maria de Lourdes Setsuko Ayrizono, Raquel Franco Leal

**Affiliations:** School of Medical Sciences, University of Campinas, Campinas, São Paulo, Brazil

**Keywords:** case report, intestinal obstruction, rectal adenocarcinoma, surgery, ulcerative colitis

## Abstract

**Introduction and importance:**

Ulcerative colitis (UC) is a chronic inflammatory disease associated with an increased colorectal cancer risk 8–10 years after diagnosis. Total proctocolectomy is usually elective and indicated for high-grade dysplasia or adenocarcinoma detected during surveillance colonoscopy. This report describes an unusual urgent case involving total mesorectal excision in a UC patient with intestinal obstruction due to a rectal lesion suspected of malignancy.

**Case presentation:**

A 38-year-old woman with UC presented with acute intestinal obstruction due to a rectal stenosis suggestive of neoplasia. She underwent total proctocolectomy with total mesorectal excision and terminal ileostomy. Histopathology revealed moderately differentiated rectal adenocarcinoma. The patient recovered well and initiated adjuvant chemotherapy, which was changed owing to radiological evidence of peritoneal implants detected 1 year after surgery. She has no further evidence of peritoneal involvement, as seen on a PET/CT scan performed 2.5 years after surgery.

**Clinical discussion:**

Patients with UC of more than 7 years’ duration have up to a tenfold higher risk of malignancy, underscoring the need for annual colonoscopy from the eighth year onward, particularly in pancolitis. Total proctocolectomy is indicated when dysplasia or carcinoma is detected. Performing oncologic surgery with total mesorectal excision and high vascular ligation in an emergency setting is uncommon.

**Conclusion:**

This case illustrates a rare malignant rectal transformation in long-standing UC, presenting with acute intestinal obstruction. Oncologic total proctocolectomy was feasible as an urgent procedure, given the patient’s condition and the colorectal surgery team’s availability in the emergency unit. The case emphasizes the importance of continuous surveillance and individualized surgical decision-making in emergency settings.

## Introduction

Ulcerative colitis (UC) is an inflammatory bowel disease (IBD) characterized by continuous mucosal inflammation extending proximally from the rectum through the colon. Its multifactorial etiology involves genetic, environmental, and gut microbiota factors. UC has a bimodal age distribution, peaking at 15–30 and 50–80 years^[^1,2^]^. The risk of colorectal cancer (CRC) increases markedly after 8–10 years of disease and is associated with male sex, younger age at diagnosis, family history of CRC, primary sclerosing cholangitis, colonic strictures, and pancolitis^[^2,3^]^. CRC surveillance is recommended after 8 years of IBD, and total proctocolectomy has historically been recommended for dysplasia because of the risk of synchronous or metachronous lesions^[^4^]^.

HIGHLIGHTS

- A rare case of rectal obstruction due to cancer in long-standing ulcerative colitis.
- A malignancy occurred despite recent negative colonoscopic findings.
- Total mesorectal excision achieved full oncologic resection in an emergency setting.

Total proctocolectomy with total mesorectal excision is the standard treatment for most patients with UC-associated rectal cancer, removing at-risk colonic and rectal mucosa. Although long-term outcomes are generally acceptable, prognosis depends on tumor stage, lymph node involvement, and resection margins^[^5^]^. Emergency oncologic resection for obstructive rectal cancer in patients with UC is rarely reported, and evidence regarding its management remains scarce. In this setting, diversion or endoluminal stenting may provide decompression or temporary stabilization before definitive resection, although stenting carries risks of migration and perforation, particularly in inflamed tissue^[^5^]^.

This report describes a patient with long-standing UC who developed an obstructing rectal adenocarcinoma requiring emergency surgery, for whom resection was feasible.

## Methodology

This case report followed the 2025 SCARE (Surgical Case Report) protocol for surgical case reporting^[^6^]^ and was approved by the local Research Ethics Committee (CAAE: 91917525.7.0000.5404). The patient provided informed consent for publication of the clinical case and related images. The authors declare that no artificial intelligence tools were used in the preparation, analysis, interpretation, or writing of this manuscript.

## Case report

A 38-year-old female patient was admitted to the emergency department with progressive abdominal pain, abdominal distension, persistent hematochezia, and complete cessation of stool and gas passage.

Diagnosed with UC in 2012 after presenting with hematochezia (Montreal E2), the patient had previously received mesalamine, azathioprine, and adalimumab during outpatient follow-up care. She denied smoking, alcohol abuse, comorbidities, previous surgeries, allergies, or a family history of CRC or IBD.

In 2020, during the COVID-19 pandemic, she developed sporadic lower abdominal pain relieved by bowel movements but did not seek care, was lost to follow-up, and discontinued treatment. Three years later, she returned with occasional abdominal cramps and tenesmus. Colonoscopy revealed diffuse colonic pseudopolyps and rectal stenosis extending more than 8 cm from the anal verge, hindering but not preventing instrument passage, with normal-appearing mucosa to the terminal ileum (Fig. 1A–F). Biopsies showed active chronic ulcerative proctitis with crypt microabscesses and regenerative epithelial atypia, without malignancy (Fig. 1G–H). She resumed follow-up and biologic therapy. In early 2024, she presented with ten bloody bowel movements per day, crampy abdominal pain, vomiting, and unquantified weight loss. An abdominal X-ray showed diffuse colonic distension (Fig. 1I–K), but she left before the evaluation was completed.

**Figure 1.**
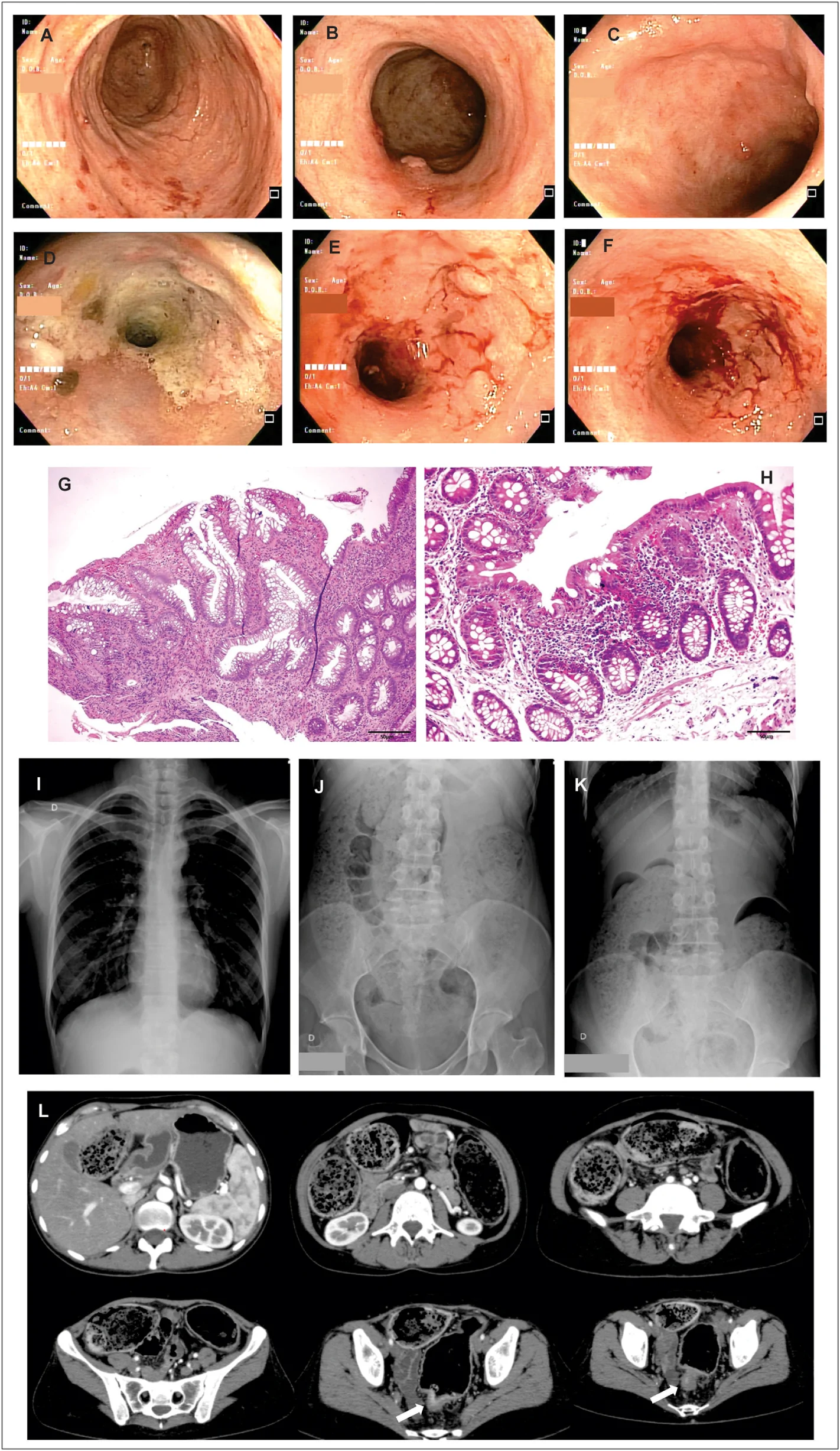
Endoscopic and histopathological findings during the patient’s follow-up demonstrated structural and inflammatory changes in the distal rectum, and radiological findings obtained in the emergency setting. (A–C) Colonic mucosa with enanthema and whitish scarred areas, in addition to pseudopolyps. (D–F) Rectal stenosis, 8 cm from the anal verge, with pseudopolyps. (G) Glandular architectural distortion (100× magnification). (H) Histological finding consistent with active chronic proctitis with inflammatory infiltrate (200× magnification). (I) Chest X-ray. (J, K) Abdominal X-rays in the supine (J) and upright (K) positions, showing diffuse colonic distension with large intraluminal fecal content. (L) Cross-sectional abdominal computed tomography images in the arterial phase, showing abrupt narrowing of the rectum up to the rectosigmoid transition (white arrows).

One month later, she returned with worsening abdominal pain, cessation of the passage of gas and stool, and persistent hematochezia. She was afebrile and hemodynamically stable, with a distended, tympanic abdomen and diffuse pain without peritonitis. Rectal examination revealed a stenosis 8 cm from the anal verge. Laboratory tests showed normocytic, normochromic anemia (Hb 10 g/dL), prealbumin 25 mg/dL, albumin 4 g/dL, and CEA 1.16 ng/mL. CT (Fig. 1L) showed colonic dilation, diffuse parietal hyperenhancement and thickening, and abrupt narrowing from the mid-rectum to the rectosigmoid junction, suggesting inflammation with subocclusion. Rectal neoplasia could not be excluded. Given the acute obstruction, urgent surgery was indicated.

During exploratory laparotomy, diffuse colonic distension and a firm tumor in the mid and upper rectum were identified without perforation (Fig. 2A and B). The lesion measured approximately 6 cm and was firmly adherent to the posterior uterine wall, although a preserved dissection plane allowed complete resection without vaginal involvement. Sparse mesocolic lymphadenopathy was observed, with no liver lesions suggestive of secondary involvement. Total proctocolectomy was performed with complete mesorectal excision and ligation of the inferior mesenteric vessels at their origin (Fig. 2C). Mesorectal dissection was carried down to the pelvic floor according to total mesorectal excision principles. The distal transection of the surgical specimen was performed in the lower rectum, 1.0 cm from the pectinate line, using a mechanical stapler, resulting in a distal margin of approximately 2 cm free of tumor macroscopically (Fig. 2D and E). Given the emergency setting, marked colonic distension, and the need for definitive oncological treatment, reconstruction was deferred. A terminal ileostomy was performed, and a suction drain was placed in the pelvis (Fig. 2F–H). No major postoperative complications occurred, such as surgical site infection, intra-abdominal abscess, postoperative bleeding, or anastomosis-related complications.

**Figure 2.**
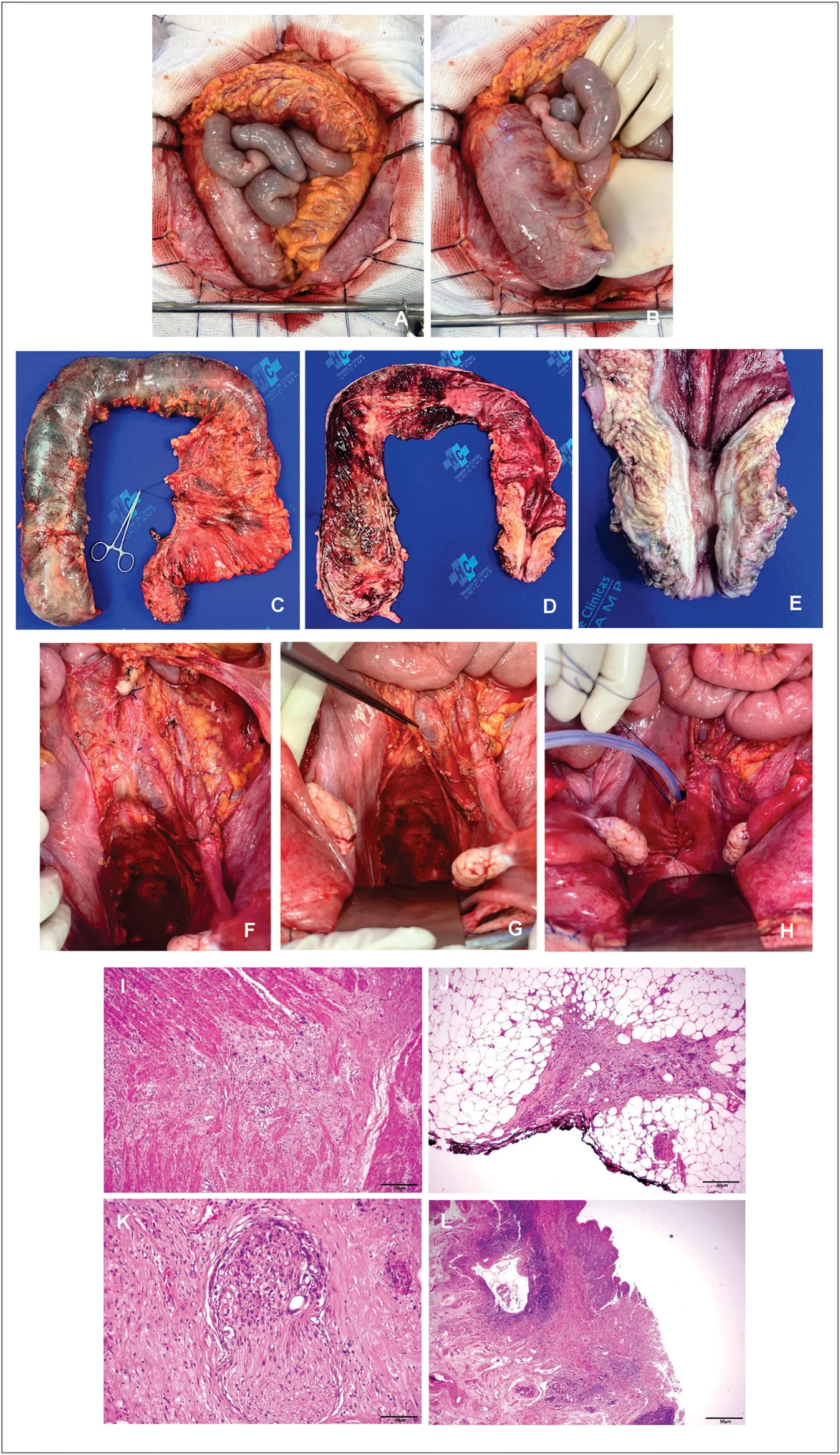
Intraoperative findings during exploratory laparotomy, surgical specimen, and histopathological analysis. (A) Distension of the entire colon. (B) Emphasis on cecal distension. (C) Surgical specimen of the total proctocolectomy with total mesorectal excision showing ligation of the inferior mesenteric vessels at their origin (suspensions repaired using a Kelly clamp). (D) Presence of enanthema throughout the mucosa, with areas of ulceration and pseudopolyps, in addition to an area of rectal stenosis. (E) Close-up photograph of the area of rectal stenosis showing the resection margin. (F) Appearance of the pelvis after removal of the surgical specimen. (G) Hypogastric nerve divided into its two branches. (H) The peritoneum of the lower abdominal cavity was used to close the pelvic cavity, with passage of a tubular drain externalized through the peritoneal closure suture toward the abdominal wall. (I) Histopathological analysis showed a moderately differentiated adenocarcinoma of the rectum (glandular tissue) invading the muscular layer, at 10× magnification. (J) Radial margin of the extraperitoneal rectum (mesorectum) was focally compromised by the neoplasm, at 200x magnification. (K) Perineural tumor invasion, at 200x magnification. (L) Distal margin of the lower rectum, showing squamous epithelial tissue without tumor infiltration, at 40x magnification.

The patient was admitted to the intensive care unit, clinically and hemodynamically stable, breathing spontaneously, and receiving ciprofloxacin and metronidazole. She required one postoperative blood transfusion for an Hb of 7.2 g/dL, without clinical sequelae. Parenteral nutrition was initiated on postoperative day 2 for paralytic ileus. Oral liquids were started on day 4 after ileostomy function resumed. C-reactive protein levels decreased from 99 mg/L on day 2 to 13 mg/L on day 5. On day 6, parenteral nutrition was discontinued, the drain removed, and the diet advanced. She was discharged on day 8 with a functioning ileostomy and good tolerance of a solid diet. Histopathology (Fig. 3I–L) revealed a moderately differentiated rectal adenocarcinoma measuring 6.0 × 5.5 × 1.2 cm, infiltrating perirectal adipose tissue to less than 1 mm from the visceral peritoneal surface (pT3), with high-grade tumor budding (Bd3). Proximal and distal margins were free of neoplasia, whereas the circumferential resection margin was focally involved. Metastases were identified in 5 of 30 lymph nodes. Initially, the patient received adjuvant capecitabine and oxaliplatin. One year after surgery, surveillance imaging (CT and PET-CT) identified pelvic peritoneal nodules (Stage IVC). The regimen was transitioned to fluorouracil, oxaliplatin, and bevacizumab. Therefore, the change in the chemotherapy regimen was due to adverse effects and the need to treat suspicious peritoneal abnormalities identified on serial radiologic reassessment.

**Figure 3.**
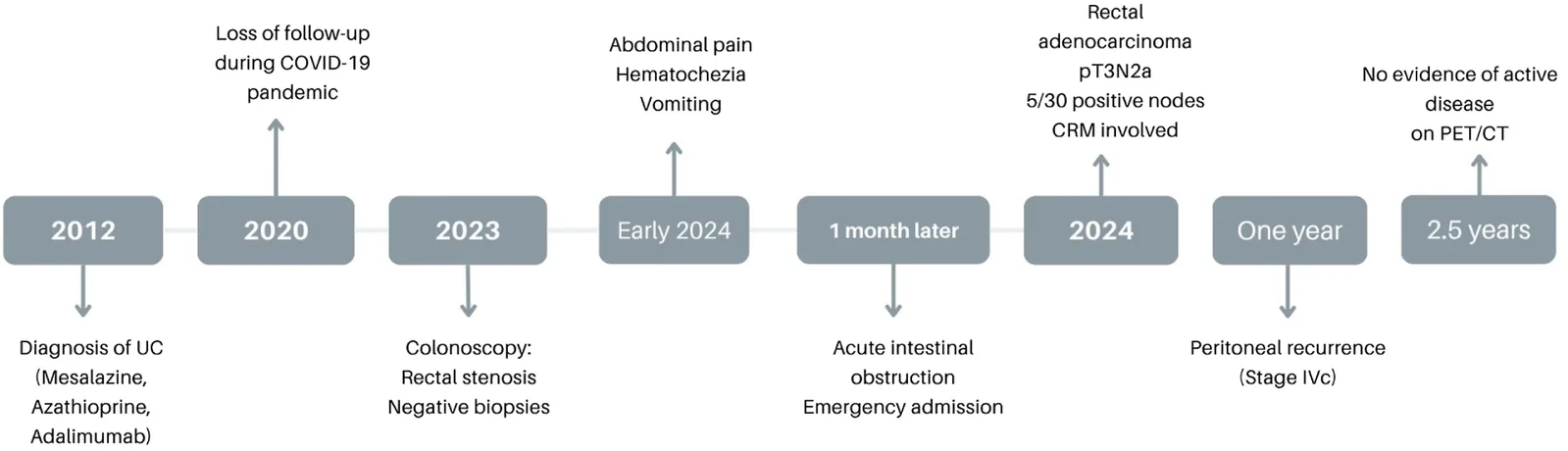
Clinical timeline of the reported case. Timeline summarizing the major clinical events from the diagnosis of UC in 2012 through the development of obstructive rectal adenocarcinoma, emergency surgical treatment, pathological findings, oncological recurrence, and a 2-year follow-up. UC, ulcerative colitis; CRM, circumferential resection margin; PET/CT, positron emission tomography/computed tomography.

The patient remains under regular coloproctology and oncology follow-up, is asymptomatic, has stable disease, and shows no evidence of active peritoneal or distant progression on PET/CT 2.5 years after surgery. She remains on maintenance fluorouracil and bevacizumab and has adapted well to the terminal ileostomy, with adequate stoma management and preserved daily activities. Before surgery, quality of life was impaired by abdominal pain, hematochezia, progressive obstruction, and activity limitations. Restorative surgery with ileal pouch-anal anastomosis was discussed with the patient; however, it is not currently planned. At present, stoma reversal is considered inappropriate, and the patient continues with a permanent terminal ileostomy to ensure oncological safety and maintain quality of life.

Despite previous biopsies showing only active chronic proctitis with regenerative atypia, persistent rectal stenosis, progressive obstruction, weight loss, and concerning imaging findings raised a strong suspicion of malignancy. Given the acute large-bowel obstruction and risk of complete obstruction or perforation, urgent surgery was favored over further investigation. Figure 3 summarizes the clinical course from UC diagnosis through obstructive rectal adenocarcinoma, emergency surgery, and oncological follow-up.

## Discussion

UC-associated rectal cancer tends to occur at a younger age than sporadic cancer and is less common than colonic cancer^[^7^]^. The patient’s age (38 years) matches the mean age reported by Radice *et al* among 18 cases–approximately 15 years younger than in sporadic cases. Although rectal adenocarcinoma is more common in males (1.4:1), the sex distribution is more balanced in UC-associated cases, particularly among patients younger than 45 years^[^8^]^.

CRC incidence in UC increases by 15–40% in pancolitis and with age and disease duration. Gorfine *et al* reported a tenfold higher malignancy risk among 756 patients with UC of more than 7 years’ duration, consistent with this case. Irregular surveillance and loss to follow-up may have delayed cancer detection and allowed progression to obstruction^[^9^]^.

Biopsies of the stenotic rectal segment showed chronic active proctitis with regenerative atypia but no malignancy, highlighting the limitations of superficial sampling in stenotic inflammatory lesions. Persistent stenosis, progressive symptoms, and concerning imaging should, therefore, raise suspicion for CRC despite inconclusive biopsies.

Intestinal obstruction as an initial manifestation of CRC in patients with UC is rare but clinically significant. Radice et al. (1998) reported more frequent bowel movements after mesorectal excision (6.5 vs. 4.2 per day) and nocturnal defecation (86 vs. 10), which may affect quality of life. They also reported a mean age of 38 years among patients undergoing surgery for UC-associated rectal cancer, matching the present case and reinforcing the younger age at presentation compared with that of sporadic CRC^[^8^]^. This pattern resembles our case, in which acute large-bowel obstruction prompted emergency surgery.

Most published cases are managed electively with full staging and multidisciplinary planning^[^10^]^. In contrast, our patient required urgent surgery, which precluded pelvic MRI, repeat biopsies, or neoadjuvant therapy. Nevertheless, total proctocolectomy with complete mesorectal excision and high vascular ligation achieved a resection in line with oncological principles; however, high-risk features such as high-grade budding may have influenced the patient’s clinical course. To our knowledge, emergency total mesorectal excision for obstructive rectal cancer in UC remains exceedingly rare.

In the present case, the decision to perform a terminal ileostomy was motivated by urgency, the presence of extensive inflammation, and the high risk of complications associated with intestinal anastomoses. In emergency settings, guidelines prioritize oncologic adequacy and patient safety. Immediate restorative procedures, such as ileal pouch-anal anastomosis, may increase the risk of postoperative morbidity, pelvic sepsis, and functional impairment; therefore, a terminal ileostomy was considered the safest option.

Adenis *et al* reported adverse outcomes, including fistulas, after ileoanal anastomosis, particularly following chemoradiotherapy. Radice et al. reported increased stool frequency and nocturnal defecation after total mesorectal excision, potentially impairing quality of life when immediate bowel restoration is not feasible^[^11^]^. Inoue et al. (2014) described a patient who underwent neoadjuvant chemoradiotherapy followed by total proctocolectomy, mesorectal resection and partial sphincter resection, and protective ileostomy, with no recurrence after 1 year, unlike our patient, who developed peritoneal implants during the same period^[^11,12^]^.

This case describes long-standing UC complicated by rectal adenocarcinoma presenting with acute bowel obstruction and requiring emergency surgery. The oncological resection was technically complete. However, factors such as focally involved margins, lymph node metastases (5/30), pT3 invasion, and high-grade tumor budding (Bd3) likely contributed to the peritoneal recurrence observed 1 year later. Re-resection was not feasible after an extensive emergency total proctocolectomy with complete mesorectal excision. Following chemotherapy, including bevacizumab, no further peritoneal implants were detected 2 years after surgery. Further studies are needed to evaluate surgical options in emergency settings, taking into account the expertise of the surgical team^[^13,14^]^.

To contextualize this case and identify comparable reports, a focused narrative literature search was conducted using combinations of the terms “ulcerative colitis”, “rectal cancer”, “rectal adenocarcinoma”, “proctocolectomy”, and “total mesorectal excision.” Table 1 summarizes reports of patients with UC who underwent total proctocolectomy with total mesorectal excision for rectal cancer, highlighting relevant clinical, pathological, and therapeutic features. To our knowledge, no published reports describe obstructive rectal cancer in patients with UC.

**Table 1:** Literature review of patients with ulcerative colitis undergoing total proctectomy with total mesorectal excision for rectal cancer.

Authors	Number of cases	Sex	Mean age	UC duration (years)	Tumor location	Histopathology and staging	Treatment
Radice *et al*^[^8^]^	18	Predominantly female	38	13.7	Rectum (10 – upper rectum; 5 – mid rectum; 3 – lower rectum)	Adenocarcinoma	Total proctocolectomy with ileoanal anastomosis (15); Total proctocolectomy with terminal ileostomy (3); Adjuvant chemotherapy (4); Adjuvant radiotherapy (5)
						Stage I (T1/T2N0M0): 12; Stage II (T3/T4N0M0): 1; Stage III (T1/T2/T3N + M0): 5	
Inoue *et al*^[^12^]^	1	Female	59	24	Lower rectum (2 cm from anal verge)	Moderately differentiated adenocarcinoma T3N2M0	Neoadjuvant chemoradiotherapy followed 8 weeks later by total proctocolectomy with ileoanal anastomosis
Gorfine *et al*^[^9^]^	14	Predominantly male	43.5	21.2	Rectum	Adenocarcinoma	Total proctocolectomy with ileoanal anastomosis (7); Total proctocolectomy with terminal ileostomy (7); Adjuvant chemotherapy (5); Adjuvant radiotherapy (2)
						Stage I: 9; Stage II: 2; Stage III: 3	
Adenis *et al*^[^11^]^	1	Female	50	15	Rectum	Poorly differentiated adenocarcinoma	Neoadjuvant radiotherapy + total proctocolectomy with terminal ileostomy^a^ + adjuvant chemotherapy
Remzi *et al* 2002	26	Predominantly male	–	18.6	Rectum	Adenocarcinoma	Total proctocolectomy with ileoanal anastomosis (26); Adjuvant chemotherapy and radiotherapy (7)
						Stage 0: 7; Stage I: 9; Stage II: 3; Stage III: 7	

^a^ Initially, total proctocolectomy with ileoanal anastomosis and protective ileostomy was converted to a terminal ileostomy after 7 months due to an ileovaginal fistula.

## Conclusion

This case describes a rare presentation of rectal adenocarcinoma in long-standing UC, presenting with acute obstruction and requiring emergency surgery without definitive preoperative histological confirmation. Despite a focally involved circumferential resection margin, pT3 disease, positive lymph nodes, and subsequent peritoneal recurrence treated with chemotherapy, the patient achieved a favorable long-term outcome. The case highlights diagnostic challenges despite negative biopsies. Strengths include the unusual presentation, detailed surgical description, and 2-year follow-up; limitations include the single-case design and incomplete preoperative staging.

## Data Availability

All the data of this case report is available within the manuscript.
